
JOURNAL INFORMATION
==============================
NLM Title Abbreviation: J Diabetes Investig
Journal ID: J Diabetes Investig
NLM Journal ID: 101520702
Journal ID: JDI

Journal of Diabetes Investigation

ISSN: 2040-1116
EISSN: 2040-1124
Publisher: Wiley

ARTICLE INFORMATION
==============================
PMCID: PMC4906257
DOI: 10.1111/jdi.12508
Article ID: JDI12508
Article version: 1
Subjects: Plenary Lectures; Abstracts from The 7th Scientific Meeting of the Asian Association for the Study of Diabetes and the Annual Scientific Meeting of the Hong Kong Society of Endocrinology, Metabolism and Reproduction, 21‐22 November 2015, Hong Kong, China; Plenary Lectures

Plenary Lectures

Electronic publication date: 2016 Jun 14
Print publication date: 2016 May
Volume: 7
Issue: Suppl 2
Issue title: Abstracts from The 7th Scientific Meeting of the Asian Association for the Study of Diabetes and the Annual Scientific Meeting of the Hong Kong Society of Endocrinology, Metabolism and Reproduction, 21‐22 November 2015, Hong Kong, China
First page: 9
Last page: 10
Copyright: © 2016 The Authors. Journal of Diabetes Investigation published by Asian Association for the Study of Diabetes (AASD) and John Wiley & Sons Australia, Ltd
License: This is an open access article under the terms of the Creative Commons Attribution License, which permits use, distribution and reproduction in any medium, provided the original work is properly cited.
License URL: https://creativecommons.org/licenses/by/4.0/

Page count: 2
source-schema-version-number: 2.0
component-id: jdi12508
cover-date: May 2016
details-of-publishers-convertor: Converter:WILEY_ML3GV2_TO_NLMPMC version:4.9.1 mode:remove_FC converted:14.06.2016

==============================
JDI Journal of Diabetes Investigation Open access
Official Journal of the Asian Association for the Study of Diabetes

### Plenary Lecture 1

### The Yutaka Seino distinguished leadership award presentation and lecture

W. Fujimoto
*School of Medicine, University of Washington, Washington, DC, USA*

Prof. Wilfred Y. Fujimoto, M.D., is originally from Hilo, Hawaii, and received the BA and MD degrees from Johns Hopkins University. Post-graduate training was at Columbia Presbyterian Medical Center, the National Institutes of Health, and the University of Washington. He was appointed to the faculty of the University of Washington in 1970 and Visiting Professor at Jichi Medical University in 2002.

Prof. Fujimoto was Principal Investigator of the "Japanese American Community Diabetes Study" which showed the high prevalence of type 2 diabetes in Japanese Americans and the relationship of lifestyle and biologic factors in the development of this disease. He was also closely involved in planning the "Diabetes Prevention Program" and chaired the Recruitment and Retention Committee of this landmark study. Dr. Fujimoto is an original member of the Organizing Committee of the International Diabetes Federation-Western Pacific Region Asia-Pacific Diabetes Epidemiology and Education Training Course. He has authored or co-authored over 300 research papers, reviews, and book chapters and has given numerous lectures locally, nationally, and internationally.

In recognition of his research in Japanese Americans, he received the Kelly West Award for Outstanding Achievement in Epidemiology from the American Diabetes Association. Other awards have been the Robert H. Williams-Rachmiel Levine Award from the Western Metabolism Club, the Lifetime Achievement Recognition Award from the American Diabetes Association-Washington Area, the Community Visionary Award from International Community Health Services in Seattle, and the Per Bjorntorp/Jean Vague Lecture Award from the International Chair on Cardiometabolic Risk.

Canary in the coal mine: Coal miners carried caged canaries down into coal mine tunnels with them to detect dangerous gases such as methane or carbon monoxide. The gases would kill the canary before killing the miners, thus providing a warning to exit the tunnels immediately.

The Seattle Japanese American Community Diabetes Study (JACDS) began as a small pilot study in 1978 and in 1983 expanded into a longitudinal community-based study with the objective of elucidating the etiology and pathophysiology of type 2 diabetes among Japanese Americans living in the Seattle area. Most participants were followed for 10 years, with the last examination conducted in 2000. This epidemiological research demonstrated the high prevalence of diabetes among second- and third-generation Japanese Americans, about fourfold greater than had been reported for Japanese in Tokyo at the time of the pilot study. We know today that type 2 diabetes is more prevalent among many Asian American ethnicities and that there is a growing epidemic of this disease in Asia. Hence Japanese Americans were figuratively speaking "canaries in the coal mine" as a harbinger of type 2 diabetes in persons of Asian origin.

Since migrant Japanese populations in not only the USA but also in Brazil have a higher prevalence of type 2 diabetes than native Japanese, this suggested an interaction of lifestyle and genetic predisposition in the etiology of type 2 diabetes. The JACDS explored this in a metabolically based epidemiological study that included extensive assessments of insulin sensitivity, insulin response, and adiposity with the latter including measurements of body fat distribution by both anthropometry and computed tomography. The importance of visceral adiposity as a risk factor for abnormal glucose tolerance, hypertension, coronary heart disease, the metabolic syndrome, and insulin resistance was shown. In conjunction with an examination of diet and physical activity patterns, the result was a clearer understanding of the etiology and pathogenesis of type 2 diabetes in Japanese Americans. We proposed that a lifestyle that fostered increased weight gain, especially in the visceral adipose depot, promoted the development of insulin resistance, which in turn exposed an underlying reduced β-cell reserve in susceptible individuals, resulting in glucose intolerance and, eventually in many, the development of diabetes. We as well as others have suggested that populations of Asian origin may generally have reduced β-cell reserve. We demonstrated the efficacy of dietary and exercise interventions to delay or prevent the development of diabetes through dietary and exercise interventions in individuals identified as having impaired glucose tolerance. The lessons learned from studying migrant Japanese in Seattle may in many ways be applicable to other populations of Asian origin.

### Plenary Lecture 2

### The Masato Kasuga Award for outstanding scientific achievement presentation and lecture

N. Kubota
*Department of Clinical Nutrition Therapy, The University of Tokyo, Tokyo, Japan*

Naoto Kubota is an Associate Professor in the Department of Clinical Nutrition Therapy at the University of Tokyo where he has been a member of Department of Diabetes and Metabolic Diseases, Graduate School of Medicine since 1998. He currently serves as the Director of Department of Clinical Nutrition Therapy at the University of Tokyo Hospital.

He is a certified Physician of the Japanese Society of Internal Medicine, Bariatrician of Japan Society for the Study of Obesity, and also Specialist of the Japan Diabetes Society and Japan Society of Metabolism and Clinical Nutrition.

In 2001, Dr. Kubota's co-research with Dr. Kadowaki and other 3 members was awarded the Baelz Prize. He is also the winner of the Lilly Prize (The Japan Diabetes Society) in 2008 followed by Scientific Encouragement Award (Japan Society for the Study of Obesity, 2009), Research Encouragement Prize (The Japanese Society of Constitutional Medicine, 2011), Society Award (Japanese Society of Molecular Medicine, 2012) and Research Encouragement Prize (The Japan Endocrine Society, 2012).

He is Councilor and Director of Japan Society of Experimental Diabetes and Obesity, Councilor and Advisor of Japan Society of Metabolism and Clinical Nutrition as well as Councilor of the Japan Diabetes Society and the Japan Endocrine Society.

Clarification of molecular mechanisms of type 2 diabetes using genetic engineering techniques in mice: The development of type 2 diabetes involves both a reduction in insulin secretion and an increase in insulin resistance, but involvement of insulin resistance associated with lifestyle changes, in particular, is said to play a major role in the background of the explosive growth in numbers of type 2 diabetes patients in recent years. Insulin resistance is a pathological state in which insulin action is impaired due to a variety of causes, and understanding physiological insulin signaling mechanisms and elucidating their pathophysiological roles is essential to preventing the onset and progression of type 2 diabetes and its complications.

I have identified that the nuclear receptor PPARγ is a thrifty gene, and a key molecule linked to obesity and insulin resistance. PPARγ was later reported to be a type 2 diabetes susceptibility gene in humans. This research not only identified PPARγ's new functions, but also contributed greatly to the study of type 2 diabetes susceptibility genes in humans. Moreover, a global expression analysis that used the adipose tissues of heterozygous PPARγ-deficient mice revealed adiponectin expression was found to be high along with leptin, and I produced adiponectin knockout mice, whose analyses directly proved that adiponectin was an insulin-sensitizing hormone *in vivo*. I also clarified that a thiazolidine derivative, which is PPARγ's synthetic ligand used as a diabetes treatment drug, improved insulin resistance in an adiponectin-dependent and adiponectin-independent manner. I also discovered adiponectin's new actions that until that time had been completely unknown, namely, activating AMP kinase in the hypothalamic arcuate nucleus, increasing appetite, and suppressing energy consumption.

I took note of the insulin receptor substrate (IRS) that exists underneath insulin receptors, and has proven, through analysis of systemic and organ-specific IRS-knockout mice, that IRS was the key molecule for physiological insulin actions as well as obesity-related insulin resistance. I showed that in pancreatic beta cells IRS-2 plays a crucial role in physiological insulin actions and in their compensatory proliferation in response to insulin resistance. Meanwhile, I found that, in the liver, the existence of a functional relay between IRS-1 and IRS-2 in insulin signaling during fasting and after refeeding; IRS-1 functions primarily after refeeding and I RS-2 functions mainly during fasting and immediately after the start of refeeding. Moreover, I showed that endothelial insulin signaling mediates insulin-stimulated capillary recruitment and increase of interstitial insulin concentrations and, as a consequence, facilitates glucose uptake by the skeletal muscle. Thus, treatment directed at improving insulin signaling in the endothelial cells may serve as a therapeutic strategy for ameliorating skeletal muscle insulin resistance. Recently I have been investigating selective insulin resistance in the liver, hoping that it could lead to the elucidation of its molecular mechanism.

© 2016 The Authors. Journal of Diabetes Investigation published by
Asian Association for the Study of Diabetes (AASD) and John Wiley & Sons Australia, Ltd
This is an open access article under the terms of the Creative Commons Attribution License, which permits use, distribution and reproduction in any medium, provided the original work is properly cited.

### Plenary Lecture 3

### The Pan Xiaoren distinguished research award for epidemiology of diabetes in Asia presentation and lecture

J. C.-N. Chan

*Department of Medicine and Therapeutics, The Chinese University of Hong Kong, Hong Kong, China*

Prof. Juliana Chan is Professor of Medicine and Therapeutics, Director of the Hong Kong Institute of Diabetes and Obesity and co-director of the International Diabetes Federation Centre of Education at the Chinese University Hong Kong, Prince of Wales Hospital. She is also the Chief Executive Officer of the Asia Diabetes Foundation. She graduated from Liverpool University, United Kingdom, with accreditations in Endocrinology and Clinical Pharmacology.

In 1995, she established the Hong Kong Diabetes Registry to first report the Asian phenotypes characterized by low/normal body mass index, visceral obesity, early age of diagnosis driven by high prevalence of childhood obesity and gestational diabetes and a propensity for developing renal disease and cancer. Using cohorts, databases and biobanks, her team has contributed to the global discovery of chromosomal regions and novel genes for type 2 diabetes. In 2007, she developed the web-based Joint Asia Diabetes Evaluation (JADE) Program incorporating risk algorithms, personalized reporting, regular feedback and decision support for quality improvement and research purposes. She has supervised 50 postgraduate students and fellows, co-authored 500 peer-reviewed articles and 20 book chapters and regularly organizes postgraduate courses and international meetings to promote scientific training and professional education. She is a leading investigator in large-scale multiomic projects supported by the US National Institute of Health and Hong Kong Government in diabetes. She is a consultant to the Hong Kong Government, multinational companies, World Health Organization and International Diabetes Federation on the subject of diabetes. She has been interviewed by the *Lancet* on the CUHK-PWH diabetes care model, and awarded the China Women Physicians Association Intercontinental Innovation Award in Clinical Research.

From epidemiology to personalized medicine in diabetes: With increasing globalization and East-West exchanges, Hong Kong witnessed an explosion of diabetes in the early 1990s with a disproportionate burden among the young and middle aged. Based on clinical observations and using large cohorts, databases and biobanks, we first reported the Asian phenotypes characterized by normal body weight with increased abdominal adiposity. The increased risk of gestational diabetes combined with exposure to poor nutrition in utero and overnutrition in later life has led to high prevalence of childhood obesity and young onset diabetes, affecting 1 in 5 adult patients. Apart from phenotypic heterogeneity, long disease duration and treatment gaps have put these young subjects at high risk of premature mortality and multiple morbidities. Genetic, environmental and psychosocial-behavioral factors interact in a complex manner to increase the risk of renal disease and cancer as leading causes of death. Using linkage analysis, genome-wide association studies and multiomic analysis, we have discovered novel loci for young onset diabetes and its complications which are implicated in developmental biology, beta cell metabolism and cell signalling pathways. By adopting the principles of clinical trials using protocols to facilitate measurements and monitoring, we further demonstrated the benefits of using task delegation, information technology, risk stratification and decision support on control of cardiometabolic risk factors, hospitalizations, morbidities and mortality. While these epidemiological discoveries are highly applicable to many populations undergoing rapid transition in developing areas, the combined use of molecular markers and care protocols provides a promising, sustainable and personalized solution for prevention and control of diabetes.

### Plenary Lecture 4

### President's lecture

K. Lam

*Department of Medicine, The University of Hong Kong, Hong Kong, China*

Prof. Karen Lam is Chair Professor in Medicine, Director of the Research Centre in Heart, Brain, Hormone & Healthy Aging, Clinical Director of the State Key Lab of Pharmaceutical & Biotechnology and Chairman of the Clinical Trial Centre at the University of Hong Kong. She is also the Department Head and Chief of Endocrinology at the University Department of Medicine, Queen Mary Hospital.

Prof. Lam was the Founding President of Diabetes Hong Kong (Honorary President since 2014), a past president of the Hong Kong Society of Endocrine, Metabolism and Reproduction, and past Chairman of two specialty boards of the HK College of Physicians – Advanced Internal Medicine and Endocrinology, Diabetes & Metabolism. She is currently the associate editor/editorial board member of several international peer-reviewed journals in diabetes and endocrinology.

Prof. Lam has published extensively on clinical, basic and translational research in diabetes and endocrinology, including publications in *Diabetes, Diabetes Care, Diabetologia*, *JCEM*, *Hypertension, ATVB, Circulation, Annals of Internal Medicine*, *Endocrinology, JBC, PNAS, JCI* and *Lancet*. Her current research focuses on the role of adipokines and hepatokines in diabetes and other obesity-related cardiometabolic disorders. Her team has established two large cohorts, the CRISPS and HKW Diabetes Registry, for the study of genetic and environmental determinants of diabetes and its related medical problems.

Insights on the roles of adipokines in clinical diabetes: The adipose tissue, previously considered as an inert energy store, is now recognized as an important endocrine organ that secretes a variety of bioactive peptides, known as adipokines or adipocytokines. Since the identification of leptin as a fat-derived hormone, an increasing number of adipokines have been identified through gene expression profiling and proteomic techniques, and shown to impact on glucose and lipid metabolism, energy homeostasis and inflammation. Dysregulated secretion of various adipokines has been demonstrated in the obese state, leading to chronic low-grade systemic inflammation and insulin resistance which, at least in part, explain why obesity is responsible for 58% of the cases of diabetes and 21% of ischemic heart disease worldwide. Of the known adipokines, adiponectin and adipocyte fatty acid binding protein (A-FABP) have the highest circulating levels in humans and are biomarkers predictive of type 2 diabetes, with increased risk being conferred by low adiponectin or high A-FABP levels. On the other hand, we have shown that, in obese individuals, the adipose tissue becomes one of the major sources of fibroblast growth factor 21 (FGF21), a hormone generally held to be a hepatokine. Beneficial metabolic effects of FGF21 have been demonstrated in animal studies and more recently, in humans, in part mediated through inducing the secretion of adiponectin. Paradoxically, high levels of FGF21 are found in obesity and type 2 diabetes, suggesting the presence of FGF21 resistance, analogous to leptin resistance in obesity. Recent studies have attributed this FGF21 resistance to adipose tissue inflammation and changes in microRNA expression, demonstrated in animal and human studies. High FGF21 levels have been found to predict the development of type 2 diabetes and diabetic nephropathy, whereas reduced FGF21 levels are found in autoimmune diabetes. The methodological difficulties that limit the clinical use of some adipokines, such as RBP-4 and angiopoietin-like-protein 4, as biomarkers in clinical diabetes and diabetic complications, will be reviewed. The relative contribution of the adipokines which have been implicated in glucose metabolism, with regard to the prediction and treatment of clinical diabetes, will be discussed.

© 2016 The Authors. Journal of Diabetes Investigation published by Asian Association for the Study of Diabetes (AASD) and John Wiley & Sons Australia, Ltd
